# Special Issue “Protein Methyltransferases in Human Health and Diseases”

**DOI:** 10.3390/ijms27167222

**Published:** 2026-08-13

**Authors:** Byron Baron

**Affiliations:** Centre for Molecular Medicine and Biobanking, University of Malta, MSD2080 Msida, Malta; byron.baron@um.edu.mt

Protein methylation is becoming more prominently recognised as a post-translational modification (PTM) that regulates diverse aspects of cellular biology through the coordinated activity of over 90 protein methyltransferases (PMTs). These enzymes catalyse the transfer of methyl groups to most commonly lysine and arginine residues in both histone and non-histone proteins, although the former are much better characterised [1]. Lysine residues may undergo mono-, di-, or trimethylation (Kme1, Kme2, Kme3), whereas arginine residues may be mono-methylated or dimethylated in either symmetric or asymmetric configurations (Rme1, sRme2, aRme2) [2]. This generates a huge diversity of chemically distinct methylation states that are spatially and temporally regulated [3]. Together, these modifications influence protein activity, intermolecular interactions, subcellular localisation, and protein stability, thereby shaping numerous physiological and pathological processes [4]. However, despite the widespread occurrence of protein methylation throughout the human proteome, the biological functions and substrate specificity of many PMTs remain poorly understood. This knowledge gap is particularly relevant to cancer and neurodegenerative disorders, where aberrant methylation contributes to disease progression and therapeutic resistance. Further complexity arises from the extensive crosstalk between methylation and other PTMs. Nevertheless, progress has been slow due to the limited availability of robust analytical tools, selective chemical probes, and comprehensive approaches for systematically characterising methyltransferase substrates and functions. This Special Issue brings together recent advances spanning mechanistic studies of protein methyltransferases, computational and structural insights into enzyme function, therapeutic inhibitor development, and disease-specific investigations, highlighting the progression from fundamental enzymology to translational applications in human disease.

Understanding the molecular mechanisms underlying methyltransferase activity represents a fundamental step toward deciphering the biological functions of protein methylation and exploiting these enzymes therapeutically. In this context, computational approaches have emerged as powerful tools for providing atomistic insights into enzyme catalysis that are often inaccessible through experimental approaches alone. Recent advances in quantum chemical calculations and molecular dynamics simulations have enabled detailed characterisation of methyl group transfer reactions, substrate recognition, catalytic residues, and conformational dynamics within S-adenosylmethionine (SAM)-dependent methyltransferases [5]. Computational strategies, including quantum chemical cluster approaches and hybrid quantum mechanics/molecular mechanics (QM/MM) methodologies, provide valuable information regarding transition states, reaction pathways, and the contribution of surrounding molecular environments to enzyme function [6]. Beyond improving fundamental understanding, these approaches can guide the rational design of selective inhibitors and engineered enzymes with altered specificity, establishing an important link between mechanistic enzymology and future therapeutic development.

Building upon this mechanistic foundation, therapeutic targeting of protein methyltransferases represents an emerging strategy for addressing diseases driven by aberrant epigenetic regulation, particularly cancer. Protein lysine methyltransferases (PKMTs) and protein arginine methyltransferases (PRMTs) regulate a diverse range of cellular processes through their ability to modify both histones, thereby regulating chromatin accessibility and gene transcription, and a wide range of non-histone proteins involved in cellular signalling and regulatory pathways [7]. As a result, the dysregulation of these enzymes is increasingly associated with tumour progression, treatment resistance, and disease recurrence [8]. Consequently, the development of small-molecule inhibitors targeting these enzymes has progressed substantially, with several compounds reaching clinical trials. However, the translation of promising preclinical findings into effective clinical therapies remains challenging due to limitations related to specificity, potency, toxicity, and the complexity of methyltransferase-dependent regulatory networks. Addressing these challenges requires a more comprehensive understanding of enzyme structure, catalytic mechanisms, substrate interactions, and the biological contexts in which individual methyltransferases contribute to disease progression [9]. Such knowledge will be essential for the development of next-generation inhibitors with improved therapeutic potential.

The importance of protein methyltransferases as therapeutic targets is further illustrated by their involvement in specific cancer contexts, where methylation-dependent signalling pathways contribute to tumour initiation and progression. Breast cancer provides a particularly relevant example, given its molecular heterogeneity and the diverse regulatory mechanisms that influence tumour behaviour and treatment response. Protein arginine methyltransferases, particularly PRMT1 and PRMT5, have emerged as important regulators of breast cancer biology through their ability to methylate both histone and non-histone substrates, thereby influencing transcriptional regulation, signalling pathways, and cellular phenotypes associated with malignancy [10]. Understanding the context-dependent functions of these enzymes across different breast cancer subtypes is essential for identifying vulnerabilities that can be therapeutically exploited [11]. The continued investigation of PRMT-mediated regulation highlights how disease-specific studies can transform fundamental insights into protein methylation can be translated into clinically relevant strategies for precision oncology.

Extending these concepts from target validation toward therapeutic application, the development of new methyltransferase inhibitors represents an important step towards overcoming current limitations in cancer treatment. One such challenge is the inability of many therapeutic molecules to cross the blood–brain barrier, limiting treatment options for patients with brain metastases [12]. Optimising the pharmacological properties of these compounds, particularly their ability to cross the blood–brain barrier, represents an important step towards expanding their therapeutic potential. The development and evaluation of novel compounds targeting EZH2 and associated components of the Polycomb Repressive Complex 2 (PRC2) pathway illustrate how structural optimisation can address limitations of existing inhibitors [13]. Importantly, therapeutic activity may not always arise exclusively through classical inhibition of canonical methylation events, but may also involve the modulation of broader signalling networks regulating methyltransferase function [14]. These findings highlight the complexity of targeting methyltransferases and emphasise the importance of understanding both enzymatic activity and wider cellular consequences when developing future therapies.

While cancer remains a major focus of protein methyltransferase research, increasing evidence demonstrates that these enzymes have broad biological roles extending beyond tumour-associated processes. The lysine methyltransferase SMYD5 exemplifies this diversity, contributing to developmental regulation, inflammatory responses, cellular stress adaptation, RNA-related processes, and viral transcription [15]. SMYD5 has been shown to regulate both histone substrates, including H3K36, H3K37, and H4K20 residues, and non-histone targets such as ribosomal and viral proteins, highlighting the functional versatility of protein methylation beyond chromatin regulation [16]. Its broad expression profile and involvement in multiple physiological and pathological processes emphasise the need for a more complete understanding of individual methyltransferases within specific cellular contexts. These findings reinforce a central theme across the field, that protein methyltransferases represent a highly diverse group of regulatory enzymes whose functions cannot be fully understood solely through their catalytic activity, but require integration of substrate identification, cellular localisation, biological context, and interaction networks.

Collectively, the contributions presented in this Special Issue demonstrate the remarkable breadth of contemporary protein methyltransferase research, spanning mechanistic enzymology, computational modelling, therapeutic inhibitor development, disease-specific regulation, and the expanding physiological roles of individual methyltransferases. Together, these studies illustrate a common progression from understanding catalytic mechanisms and molecular interactions, through target validation, toward the development of therapeutic strategies and clinical applications. At the same time, they highlight that the biological importance of protein methylation extends beyond oncology, influencing diverse aspects of cellular regulation and human disease.

Despite substantial advances, many fundamental questions remain unresolved. The complete repertoire of methyltransferase substrates remains incompletely defined, the functional consequences of different methylation states (including mono-, di-, and trimethylation) require further clarification, and the extensive interplay between methylation and other PTMs continues to represent a major challenge. Furthermore, improving the clinical translation of methyltransferase inhibitors will require a deeper understanding of enzyme biology, substrate recognition and specificity, and the molecular mechanisms underlying therapeutic response and resistance.

Future progress will depend on the continued integration of complementary technologies, including structural biology, computational chemistry, chemical biology, and advanced proteomic approaches. In particular, advances in quantitative mass spectrometry and functional proteomics will be instrumental in identifying novel methylation sites, discovering new methyltransferase substrates, and elucidating their roles in cellular biology and disease. Ultimately, a more comprehensive understanding of protein methyltransferases will not only advance fundamental biology but also support the development of more precise diagnostic and targeted therapeutic approaches for a wide range of human diseases.
